# Citrate shows protective effects on cardiovascular and renal function in ischemia-induced acute kidney injury

**DOI:** 10.1186/s12882-017-0546-1

**Published:** 2017-04-10

**Authors:** Anja Bienholz, Jonas Reis, Pinar Sanli, Herbert de Groot, Frank Petrat, Hana Guberina, Benjamin Wilde, Oliver Witzke, Fuat H. Saner, Andreas Kribben, Joel M. Weinberg, Thorsten Feldkamp

**Affiliations:** 1 Department of Nephrology, University Hospital Essen, University Duisburg-Essen, Hufelandstr. 55, 45147 Essen, Germany; 2 Institute of Physiological Chemistry, University Hospital Essen, University Duisburg-Essen, Hufelandstr. 55, 45147 Essen, Germany; 3 Department of Infectious Diseases, University Hospital Essen, University Duisburg-Essen, Hufelandstr. 55, 45147 Essen, Germany; 4 Department of General, Visceral and Transplant Surgery, University Hospital Essen, University Duisburg-Essen, Hufelandstr. 55, 45147 Essen, Germany; 5 Department of Internal Medicine, Division of Nephrology, V.A. Ann Arbor Health System and University of Michigan, 1150 W. Medical Center Drive, 1560C MSRB II, Ann Arbor, MI 48109-5676 USA; 6 Department of Nephrology and Hypertension, University Hospital Schleswig-Holstein, Christian-Albrechts-University, Schittenhelmstr. 12, 24105 Kiel, Germany

**Keywords:** Blood pressure, Ischemia and reperfusion, Renal clamping, Citrate

## Abstract

**Background:**

Ischemia and reperfusion (I/R) is one of the major causes of acute kidney injury (AKI). Citrate reduces hypoxia-induced mitochondrial energetic deficits in isolated proximal tubules. Moreover, citrate anticoagulation is now frequently used in renal replacement therapy. In the present study a rat model of I/R-induced AKI was utilized to examine renal protection by citrate in vivo.

**Methods:**

AKI was induced by bilateral renal clamping (40 min) followed by reperfusion (3 h). Citrate was infused at three different concentrations (0.3 mmol/kg/h; 0.6 mmol/kg/h and 1.0 mmol/kg/h) continuously for 60 min before and 45 min after ischemia. Plasma calcium concentrations were kept stable by infusion of calcium gluconate. The effect of citrate was evaluated by biomonitoring, blood and plasma parameters, histopathology and tissue ATP content.

**Results:**

In comparison to the normoxic control group bilateral renal ischemia led to an increase of creatinine and lactate dehydrogenase activity and a decrease in tissue ATP content and was accompanied by a drop in mean arterial blood pressure. Infusion of 1.0 mmol/kg/h citrate led to lower creatinine and reduced LDH activity compared to the I/R control group and a tendency for higher tissue ATP content. Pre-ischemic infusion of 1.0 mmol/kg/h citrate stabilized blood pressure during ischemia.

**Conclusions:**

Citrate has a protective effect during I/R-induced AKI, possibly by limiting the mitochondrial deficit as well as by beneficial cardiovascular effects. This strengthens the rationale of using citrate in continuous renal replacement therapy and encourages consideration of citrate infusion as a therapeutic treatment for AKI in humans.

**Electronic supplementary material:**

The online version of this article (doi:10.1186/s12882-017-0546-1) contains supplementary material, which is available to authorized users.

## Background

Acute kidney injury (AKI) is still a life threatening disease. The application of uniform definitions and classifications during the past few years has shown that even minor forms of AKI documented by minimal and totally reversible rises in plasma creatinine are accompanied by decreased patient survival and progression of chronic kidney disease [1, 2]. AKI affects patients of various medical disciplines, for example during sepsis or following surgery with ischemia characterized by a shortage in oxygen and nutrition supply being its major cause [3]. It particularly occurs in critically ill patients, e.g. after heart surgery and liver transplantation with an incidence of up to 50% [4].

Despite substantial progress in medicine in general, supportive treatment is still the only therapeutic strategy in AKI. Renal replacement therapy is one of the major options in severe cases of AKI. Kidney Disease Improving Global Outcomes (KDIGO) guidelines suggest the use of regional citrate anticoagulation instead of heparin in patients with AKI receiving continuous renal replacement therapy [5]. This suggestion is not only based on superior filter longevity [6, 7], but also due to a reduction in bleeding risks accompanied by regional anticoagulation limited to the extracorporeal circuit [7–9]. Citrate anticoagulation can lead to a number of metabolic complications (acidosis, alkalosis, hypernatremia, hypocalcemia) and should therefore only be used in centers providing the necessary expertise. However, incorporating a locally adapted protocol and adequate metabolic biomonitoring especially including ionized calcium levels during treatment citrate anticoagulation can safely be used even in patients after liver transplantation [10].

In addition to increased filter longevity and decreased bleeding risks there are suggestions of direct advantages from citrate application. Citrate may suppress inflammation [9, 11] and addition of citric acid cycle metabolites including citrate itself during hypoxia and/or reoxygenation to isolated proximal renal tubules improves energetic function and recovery by driving substrate-level phosphorylation that lowers accumulated non-esterified fatty acids [12, 13]. We recently introduced a novel rodent based animal model for studying ischemia/reperfusion (I/R) AKI in vivo that incorporates intensive biomonitoring during the ischemic and early postischemic periods. Using the system we found that infusion of a substrate combination of α-ketoglutarate plus malate that was highly protective in vitro aggravated hypotension during ischemia and was not protective in vivo [14]. The methodology developed for the latter study lends itself to assessment of citrate effects since it allows for the infusion and monitoring that are necessary to employ citrate in the clinical setting. In the present studies we have used the approach to examine whether citrate provides renal protection in vivo.

## Methods

### Chemicals/Materials

Hematoxylin, ß-NADH, citrate lyse, L-lactic dehydrogenase, malic dehydrogenase, triethanolamine and citrate were obtained from Sigma-Aldrich (Steinheim, Germany). Formalin solution (4.5% and 10%, buffered) and isoflurane (Forene) were from Abbott (Wiesbaden, Germany), ketamine 10% was from Ceva (Düsseldorf, Germany), lidocaine (Xylocaine 1%) from AstraZeneca (Wedel, Germany), Ringer’s solution Macoflex N from MacoPharma International (Langen, Germany), 0.9% NaCl solution and sterile water (Aqua Ecotainer) were from Braun (Melsungen, Germany), paraffin (Paraplast Tissue Embedding Medium REF 501006) was from McCormick Scientific (St. Louis, MO), medical oxygen from Air Liquide (Düsseldorf, Germany) and heparin-sodium 25000 from Ratiopharm GmbH (Ulm, Germany). Triton X-100, zinc chloride, ammonium sulfate and magnesium sulfate were purchased from AppliChem (Darmstadt, Germany), Tris from Serva Electrophoresis (Heidelberg, Germany), Dulbecco’s Phosphate Buffered Saline (DPBS) from Invitrogen (Darmstadt, Germany) and EDTA from Merck (Darmstadt, Germany). Syringe pumps (Perfusor-Secura FT) were from Braun (Melsungen, Germany), portex catheters (0.58 mm i.d., 0.96 mm o.d.) from Smiths Medical International (Hythe, U.K.), 4–0 Vicryl sutures from Ethicon (Norderstedt, Germany), 2-ml syringes (Pico50) from Radiometer Medical (Brønshøj, Denmark), safe-lock tubes (2 ml) from Eppendorf (Hamburg, Germany) and 15 ml polypropylene tubes (Falcon tubes) from BD Biosciences (Heidelberg, Germany).

### Animals

Male Sprague Dawley rats (390–490 g) were obtained from Charles River (Sulzfeld, Germany). Animals were kept for at least one week prior to the experiments in the central animal unit of the University Hospital Essen under standardized conditions of temperature (22 ± 1 °C), humidity (55 ± 5%) and 12 h/12 h light/dark cycles with free access to food (ssniff-Spezialdiäten, Soest, Germany) and water; animals were not fasted prior to the experimental procedures.

### Anesthesia, analgesia and surgical procedure

Anesthesia with isoflurane and analgesia with ketamine was performed as described previously [14, 15]. A skin-deep incision was made along the thigh of both hind limbs after application of lidocaine (5 mg/kg s.c.). Portex catheters were placed in the exposed right femoral artery and both femoral veins as well as in the urinary bladder and fixed with 4–0 Vicryl ligatures. Following this procedure, a median abdominal laparotomy was performed along the *Linea alba*. After an acclimation period of 30 min and the first infusion period (60 min) the intestine was carefully evacuated from the abdominal cavity and both kidneys were localized. The vascular pedicle of each kidney was mobilized. In animals undergoing clamping both renal pedicles were occluded for 40 min using atraumatic mini-bulldogs (Aesculap, Tuttlingen, Germany). The intestine was replaced into the abdominal cavity and covered with moistened compresses and aluminum foil to minimize evaporation and cooling. Subsequent to the ischemic period of 40 min the microvascular clamps were removed and the kidneys thus reperfused. At the end of the reperfusion period of 180 min, including a second infusion period (45 min) the right kidney was removed. A catheter was placed in the abdominal aorta and the left kidney perfused at 100 mmHg with 40 ml isotonic NaCl solution containing 1500 I.U. heparin-sodium before being resected. Animals remained anesthetized during the whole experiment and were sacrificed by cardiac incision under deep isoflurane anesthesia.

### Study groups

**Fig. 1:**
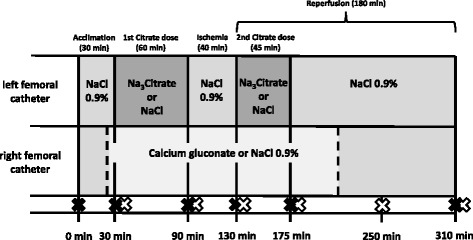
Experimental design. Infusions through each catheter during each experimental period are indicated. Both renal pedicles were atraumatically clamped during the 40 min ischemic period. In the initial study, the dose of trisodium citrate administered pre-ischemia for 60 min and during the first 45 min of reperfusion was 0.3, 0.6 or 1.0 mmol/kg/h at a rate of 5 ml/kg/h. In the normoxic and I/R control groups, 0.9% NaCl solution was infused instead at the same rates. In a follow-up study, 1.0 mmol/kg/h trisodium citrate was compared to 3.0 mmol/kg/h NaCl delivered as a 3.5% solution. Calcium gluconate solutions were administered at 2 ml/kg/h. [cross black], blood sampling, [cross white], urine sampling. Blood pressure, heart rate, respiratory rate, core body temperature, and peripheral oxygen saturation were continuously monitored during the entire experiment

The protocols used are diagrammed in Fig. 1. The initial animal study was performed with six rats per group. The following experimental groups were compared:

- Group 1: normoxic control group, sham-operation, 0.9% NaCl;
- Group 2: I/R control group, 40 min renal clamping, 0.9% NaCl;
- Group 3: I/R citrate group, 40 min renal clamping, citrate 0.3 mmol/kg/h (cumulative dose of citrate 0.525 mmol/kg ≈ 0.154 g/kg);
- Group 4: I/R citrate group, 40 min renal clamping, citrate 0.6 mmol/kg/h (cumulative dose of citrate 1.05 mmol/kg ≈ 0.309 g/kg);
- Group 5: I/R citrate group, 40 min renal clamping, citrate 1.0 mmol/kg/h (cumulative dose of citrate 1.75 mmol/kg ≈ 0.515 g/kg).

Citrate for all groups where it was used was administered for 60 min prior to the clamp and during the first 45 min of reperfusion. The dose of citrate studied was based on the use of the average dose for patients in the University Hospital Essen who receive about 0.6 mmol/kg/h citrate for anticoagulation during continuous renal replacement therapy [10]. As the amount of citrate eliminated in continuous veno-venous hemodialysis is up to 50%, the amount of citrate reaching patients circulation is most likely only about 0.3 mmol/kg/h.

The ischemic control group and the normoxic control group of rats undergoing all surgical procedures except clamping of the renal pedicles received only 0.9% NaCl solution at a rate of 5 ml/kg/h during the experimental period.

Citrate solutions were prepared from sterile 30% trisodium citrate solution (pH 4.84) available for regional anticoagulation at the University Hospital Essen (Pharmacy, University Hospital Essen). Sterile 30% trisodium citrate solution was freshly dissolved in bidistilled water to obtain final solutions containing

- 0.3 mmol citrate/3 ml (0.401 ml 30% trisodium citrate solution plus 2.599 ml bidistilled water);
- 0.6 mmol citrate/3 ml (0.802 ml 30% trisodium citrate solution plus 2.198 ml bidistilled water);
- 1.0 mmol citrate/3 ml (1.337 ml 30% trisodium citrate solution plus 1.663 ml bidistilled water).

Citrate solutions were filtered through a bacteria-tight filter (Minisart 0.2 μm; Sartorius, Göttingen, Germany) and infused with a syringe pump into the right *V. femoralis* at a rate of 3 ml/kg/h 60 min prior and 45 min after ischemia. As in the clinical setting, pH was not adjusted. There was no effect in any group of citrate infusion on arterial pH at the end of the initial infusion period before ischemia (not shown).

Corresponding Ca^2+^-solutions needed to ensure stable Ca^2+^ levels were based on 10% calcium gluconate pH 5.5–7.5 (B. Braun, Melsungen, Germany) and contained

- for animals receiving 0.3 mmol/kg/h citrate: 0.136 mmol Ca^2+^/2 ml (0.6 ml 10% calcium gluconate plus 1.4 ml bidistilled water);
- for animals receiving 0.6 mmol/kg/h citrate: 0.271 mmol Ca^2+^/2 ml (1.2 ml 10% calcium gluconate plus 0.8 ml bidistilled water);
- for animals receiving 1.0 mmol/kg/h citrate: 0.452 mmol Ca^2+^/2 ml (undiluted 10% calcium gluconate).

Calcium gluconate solutions were filtered through a bacteria-tight filter (Minisart 0.2 μm; Sartorius, Göttingen, Germany) and infused with a with a syringe pump into the left *V. femoralis* at a rate of 2 ml/kg/h. As in the clinical setting, pH was not adjusted. Infusion of calcium gluconate was started 10 min prior to infusion of citrate solutions and was continued throughout ischemia and during the first 80 min of reperfusion. Calcium gluconate dosage and infusion time were determined empirically in pilot studies.

After the initial results indicated hemodynamic benefit of citrate a follow-up study was then performed to control for citrate-independent effects of the Na^+^ load that accompanies citrate. This study consisted of:

- Group I: normoxic control group, sham-operation, 0.9% NaCl ≈ 0.77 mmol/kg/h Na^+^ (three rats);
- Group II: I/R control group, 40 min renal clamping, 0.9% NaCl ≈ 0.77 mmol/kg/h Na^+^ (four rats);
- Group III: I/R citrate group, 40 min renal clamping, citrate 1.0 mmol/kg/h (including 3.0 mmol/kg/h Na^+^) (cumulative dose of citrate 1.75 mmol/kg ≈ 0.515 g/kg) (six rats);
- Group IV: I/R high sodium group, 40 min renal clamping, 3.5% NaCl ≈ Na^+^ 3.0 mmol/kg/h (six rats).

As in the initial study animals of the I/R citrate 1.0 mmol/kg/h group received undiluted 10% calcium gluconate during time span indicated above.

Summarizing both experimental studies, three animals died due to cardiac arrest during acclimation/before induction of ischemia and catheter placement failed in two rats. Those five animals were replaced. Therefore, all animals exposed to ischemia were included in the final analysis.

### Monitoring of vital parameters

Vital parameters were assessed every 10 min starting with the beginning of the surgical procedure (Fig. 1). Systolic, diastolic and mean arterial blood pressures were continuously recorded via the femoral artery catheter that was connected with a pressure transducer (MX 960; Medex Medical, Rossendale, UK). An infusion bag containing Ringer’s solution delivered 3 ml/h to keep the catheter functional. At a mean arterial blood pressure below 70 mmHg for more than 5 min, bolus injections of 0.5 ml 0.9% NaCl solution were administered repetitively through the right femoral artery catheter up to a maximum volume of 5 ml/kg/h. Rat heart rates were determined from systolic blood pressure spikes. The core body temperature of the rats was continuously monitored using a rectal sensor and maintained at 37.6 ± 0.2 °C by means of an underlying thermostated operating table and by coverage with aluminum foil. The breathing rate was determined based on ventilation movements in 10-min intervals.

Urine containers were changed after 30 min, immediately before ischemia, after 40 min of ischemia, after 45, 120 and 180 min of reperfusion (Fig. 1).

### Assessment of blood and plasma parameters

Using a 2-ml syringe containing 80 I.U. electrolyte-balanced heparin, blood samples (0.5 ml) were taken from the femoral artery catheter immediately after its insertion, after 30 min, immediately before ischemia, after 40 min of ischemia, after 45 min, and after 180 min of reperfusion (Fig. 1). For each blood sample animals were replaced with 0.5 ml 0.9% NaCl solution via the femoral artery. For determination of arterial oxygen and P_CO2_, oxygen saturation, pH, acid–base status, hemoglobin concentration and hematocrit, electrolytes (Na^+^, K^+^, Cl^−^, Ca^2+^), metabolic parameters (lactate, glucose) and osmolality, a blood gas analyzer equipped with additional electrodes was used (ABL 715; Radiometer, Copenhagen, Denmark).

Blood plasma was obtained by centrifugation (3,000 × g for 15 min at 25 °C) and stored at 4 °C until its use (within 4 h). The plasma activity of lactate dehydrogenase (LDH) served as a general indicator of cell injury, while plasma creatinine (pCrea) was measured as an indicator for AKI. Plasma levels were determined spectrophotometrically by a fully automated clinical chemistry analyzer (Vitalab Selectra E; VWR International, Darmstadt, Germany).

### Determination of tissue ATP

The caudal poles of both kidneys were cut off at the end of reperfusion, immediately before kidney removal, stored in safe-lock tubes (2 ml) containing 1 M perchloric acid in an extracellular buffer for deproteinization [16], were mixed vigorously and immediately frozen in liquid nitrogen. Samples were kept frozen at −80 °C. Tissue ATP content was measured using a luciferase-driven bioluminescence assay (ATP Bioluminescence Assay Kit CLS II, Roche, Mannheim, Germany). After thawing, samples were diluted in buffer containing 100 mM Tris and 4 mM EDTA (pH 7.75) and mixed immediately with luciferase reagent. Light emission was detected at 550 nm by a luminometer (Berthold Detection Systems, Pforzheim, Germany). ATP content was normalized for tissue protein content as determined according to Lowry [17].

### Histopathological evaluation of the ischemia-reperfusion injury of the kidney

For histological examination the left blood-free kidney was sliced in half and fixed in formalin (10%, neutral buffered) for 24–48 h. Afterwards, it was embedded in paraffin and cut on a rotary microtome in serial sections of 2 μm thickness. Tissue sections were mounted on slides and stained with hematoxylin-eosin. Histopathological changes were evaluated in a blinded fashion based on the following criteria: 1. Blood content in glomeruli; 2. shrunken or swollen glomeruli; 3. loss of brush border; 4. shrunken or swollen tubular epithelial cells; 5. intraluminal casts and 6. indicators of cell death (loss of nuclei).

### Determination of citrate in plasma and urine

Citrate was enzymatically converted into ß-NAD which was detected at 37 °C with a Hitachi F-2500 fluorescence spectrophotometer (Düsseldorf, Germany) (emission: 471 nm; absorption: 353 nm).

In detail, 1 ml assay buffer (50 mM triethanolamine, 10 mM MgSO_4_, 5 mM EDTA, 8.340 ml/l L-lactic dehydrogenase, 1.628 ml/l malic dehydrogenase, pH = 7.4) was incubated in cuvettes in a light protected water bath (Lauda ecoline 003 with thermostat E100, Lauda-Königshofen, Germany) for 10 min. 10 μl citrate standard and samples were added to the cuvettes with 10 min of further incubation. Subsequently, baseline emission was measured. Immediately after the measurement 10 μl citrate lyse enzyme solution (32.6 g/l citrate lyse, 10 mM triethanolamine, 0.3 mM zinc chloride, 545 mM ammonium sulphate) was added. Emission was measured again following 60 min of incubation.

### Determination of plasma osmolality by cryoscopy

Plasma osmolality was directly determined by cryoscopy using an electronic semi-micro osmometer (Typ M, Knauer, Berlin, Germany).

In order to avoid excess amounts of blood sampling that could affect the experimental outcome, changes of osmolality were assessed in a separate study comparing three animals per group.

- Group I (treatment analog Group 2): I/R control group, 40 min renal clamping, 0.9% NaCl;
- Group II (treatment analog Group 5): I/R citrate group, 40 min renal clamping, citrate 1.0 mmol/kg/h (cumulative dose of citrate 1.75 mmol/kg ≈ 0.515 g/kg).

Animals underwent the exact same treatment as described above including biomonitoring without acquirement of blood samples except for two time points: 1. directly before ischemia following the first 60 min of citrate infusion; 2. after 100 min of reperfusion, as animals undergoing ischemia and receiving 1.0 mmol/kg/h citrate showed a peak in blood pressure at this time point in the initial experimental study.

### Statistics

Experiments were performed with six animals per experimental group unless indicated otherwise. Biochemical assays were run at least in duplicate. Data are expressed as mean values ± standard error of the mean. Comparisons among multiple groups were performed using analyses of variances either for nonrecurring or for repeated measures (analysis over time) or Kruskal-Wallis test followed by Bonferroni or Dunns *post*-hoc analysis. A p-value < 0.05 was considered significant.

## Results

### Effects of citrate on markers of organ injury and tissue ATP content

**Fig. 2:**
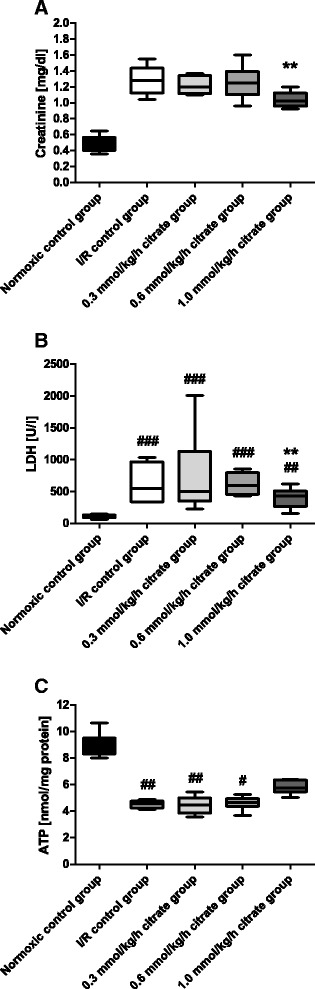
Creatinine, lactate dehydrogenase and ATP levels at the end of reperfusion. Box plots with whiskers indicating minimum and maximum, six rats per group. **a** Creatinine after 40 min of ischemia plus 180 min of reperfusion. All I/R groups were significantly different from the normoxic control group at *p* < 0.001. ** *p* < 0.01 vs. I/R control group. **b** Lactate dehydrogenase (LDH) after 40 min of ischemia plus 180 min of reperfusion. ## *p* < 0.01 and ### *p* < 0.001 vs. normoxic control group. ** *p* < 0.01 vs. I/R control group. **c** Tissue ATP content after 40 min of ischemia plus 180 min of reperfusion. # *p* < 0.05 and ## *p* < 0.01 vs. normoxic control group. No significant differences between 1.0 mmol/kg/h citrate and normoxic or I/R control groups

In the normoxic control group the pCrea and plasma LDH concentrations remained stable throughout the experiment. Kidney I/R resulted in an increase in pCrea and LDH concentrations (Fig. 2ab). Tissue ATP content was significantly decreased after I/R compared to the normoxic control group (*p* < 0.01; Fig. 2c).

Infusion of citrate had no effects on pre-ischemic values of all parameters (data not shown). Significant differences after citrate infusion compared to the I/R control group were detected in the 1.0 mmol/kg/h citrate group. In the 1.0 mmol/kg/h citrate group pCrea and LDH levels were significantly less increased after 180 min of reperfusion (*p* < 0.01 and *p* < 0.05), while tissue ATP content tended also to be higher in the 1.0 mmol/kg/h citrate group without reaching statistical significance (Fig. 2c).

**Fig. 3:**
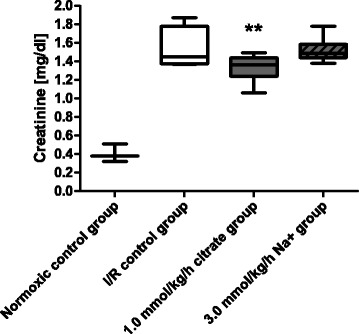
Creatinine levels for the follow-up study. The follow-up study using the same design as the initial study (see Fig. 1) was conducted to control for the citrate-independent effects of the sodium load that accompanies citrate. Box plots with whiskers indication minimum and maximum, 3–4 rats for the normoxic and I/R control groups and 6 rats per group for the 1.0 mmol/kg/h citrate and 3.0 mmol/kg/h Na^+^ groups. Creatinine after 40 min of ischemia plus 180 min of reperfusion. All I/R groups were significantly different from the normoxic control group at *p* < 0.001. ** *p* < 0.01 vs. I/R control group

In the follow-up study performed to control for citrate-independent effects of the high Na^+^ load that accompanies citrate, results for the 1.0 mmol/kg/h citrate group were confirmed, while infusion of 3.0 mmol/kg/h Na^+^ did not result in a significantly blunt the increase of pCrea levels after 180 min of reperfusion compared to the I/R control group (Fig. 3).

### Effects of citrate on histopathological changes

I/R resulted in loss of brush border, increased numbers of shrunken tubular epithelial cells and glomeruli as well as formation of intraluminal casts. No difference in histologic architecture could be detected between the I/R control group and any citrate group at this relatively short reperfusion period (not shown).

### Effects of citrate on blood pressure and other vital parameters

**Fig. 4:**
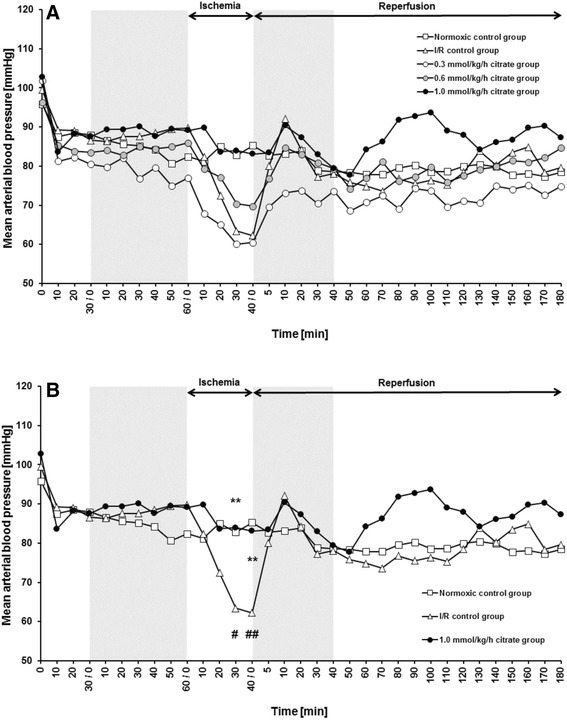
Mean arterial blood pressure. Mean arterial blood pressure was assessed every 10 min starting with the beginning of the surgical procedure. Arterial blood pressure was continuously recorded via a catheter placed in the femoral artery. Grey-shaded areas indicate the periods of infusion of trisodium citrate at indicated concentrations. Calcium levels were kept stable by corresponding infusions of calcium gluconate Normoxic and I/R control groups received only 0.9% NaCl solution. Infusion volume was 5 ml/kg/h in all animals. Values are means of six rats per group. For other experimental details see Fig. 1. **a** Mean arterial blood pressure of all experimental groups. **b** Mean arterial blood pressure of control groups and 1.0 mmol/kg/h citrate group. # *p* < 0.05 and ## *p* < 0.01 vs. normoxic control group. ** *p* < 0.01 vs. I/R control group

Baseline values of mean arterial blood pressure of all animals were around 85 mmHg after the acclimation period. In the normoxic control group, MAP slowly decreased over the course of the experiment and was 78 ± 3 mmHg (*p* = 0.02 vs. baseline) at the end of the reperfusion period. In the I/R control group, blood pressure rapidly decreased upon renal clamping showing significant difference to the MAP of the normoxic control group at 30 min (*p* < 0.05) and 40 min (*p* < 0.01) of ischemia (Fig. 4). During very early reperfusion, MAP of the I/R control group tended to be higher compared to the normoxic control group, but values of both groups converged during the course of the experiment. Pre-ischemic infusion of citrate had no immediate effect on MAP, but increased blood pressure during renal clamping in a concentration dependent manner, reaching statistical significance for the 1.0 mmol/kg/h citrate group in comparison to the I/R control group at 30 min and 40 min of ischemia (*p* < 0.01, Fig. 4b). Upon reperfusion, values of all experimental groups gradually normalized to those of the normoxic control group, but MAP of the 1.0 mmol/kg/h citrate group tended to be higher during most of the reperfusion period (Fig. 4a).

**Fig. 5:**
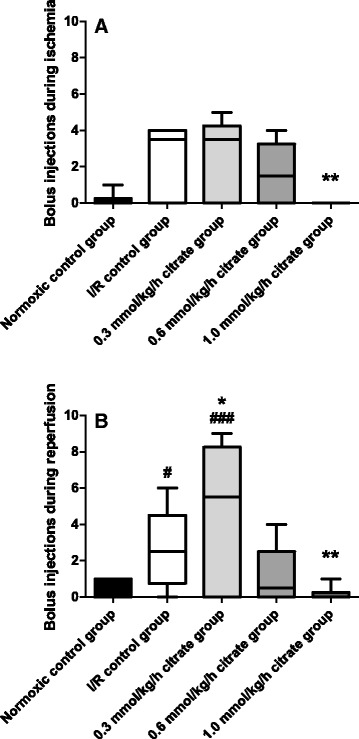
Number of bolus injections used to help support blood pressure. Rats of the I/R control group and all citrate groups were subjected to clamping of both renal vascular pedicles for 40 min followed by three hours of reperfusion as diagrammed in Fig. 1. Animals of the citrate groups received sodium citrate at indicated concentrations 60 min before and 45 min after ischemia. Calcium levels were kept stable by corresponding infusions of calcium gluconate. Normoxic and I/R control groups received only 0.9% NaCl solution. Infusion volume was 5 ml/kg/h in all animals. Additional bolus injections of 0.5 ml 0.9% NaCl were administered repetitively up to a maximum volume of 5 ml/kg/h at a mean arterial blood pressure below 70 mmHg for more than 5 min. Box plots with whiskers indicating minimum and maximum, six rats per group. **a** Bolus injections during ischemia. # *p* < 0.05 and ## *p* < 0.01 vs. normoxic control group. ** *p* < 0.01 vs. I/R control group. **b** Bolus injections during reperfusion. # *p* < 0.05 and ### *p* < 0.001 vs. normoxic control group. * *p* < 0.05 and ** *p* < 0.01 vs. I/R control group. 1 Bolus injection = 0.5 ml 0.9% NaCl

Consistent with the effects of intravenous citrate on MAP during kidney ischemia, animals of the I/R control group received significantly more bolus injections of 0.9% NaCl solution than rats of the 1.0 mmol/kg/h group during ischemia and reperfusion (1 bolus injection = 0.5 ml) (2.5 ± 0.8 vs. 0.0 ± 0.0 and 2.7 ± 0.9 vs. 0.17 ± 0.17; *p* < 0.01) (Fig. 5).

Heart rates showed high inter-individual difference with no statistical difference between experimental groups. Respiratory rate and core body temperature were not significantly changed by kidney I/R in the absence or presence of citrate in any dosage.

**Fig. 6:**
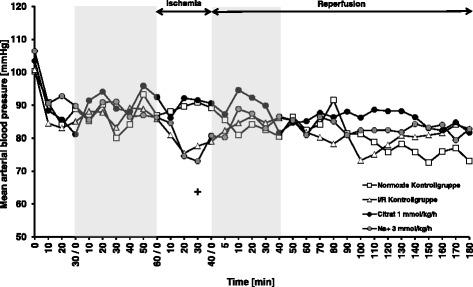
Mean arterial blood pressure in the follow-up study. The follow-up study using the same design as the initial study (see Fig. 1) was conducted to control for the citrate-independent effects of the sodium load that accompanies citrate. Mean arterial blood pressure was assessed every 10 min starting with the beginning of the surgical procedure. Arterial blood pressure was continuously recorded via a catheter placed in the femoral artery. Grey-shaded areas indicate the periods of infusion of trisodium citrate or 3.5% NaCl. Calcium levels were kept stable by corresponding infusions of calcium gluconate. Normoxic and I/R control groups received only 0.9% NaCl solution. Infusion volume was 5 ml/kg/h in all animals. Values are means of 3–4 rats for the normoxic and I/R control groups and 6 rats per group for the 1.0 mmol/kg/h citrate and 3.0 mmol/kg/h Na^+^ groups. + *p* < 0.05 vs. 1.0 mmol/kg/h citrate group

While infusion of citrate increased blood pressure during renal clamping in a concentration dependent manner, this effect was not reproduced by concentrated NaCl in the follow-up study performed to control for citrate-independent effects of the Na^+^ load that accompanies citrate (Fig. 6).

### Effects of citrate on blood pH, P_CO2_, base excess, electrolytes, and metabolic parameters

Table 1 summarizes values at baseline and at the end of the experiment. In the normoxic control group glucose and potassium levels slightly decreased over time, but remained within the physiological range. All other parameters in the normoxic control group were stable during the course of the experiment. Kidney I/R resulted in a significantly lower base excess and significantly higher potassium levels during reperfusion. No significant differences in other electrolytes, blood pH, P_CO2_, P_O2_ or metabolic parameters in comparison to the normoxic control group could be detected.

**Table 1:** Measurements of blood and plasma parameters

		Normoxic control group	I/R control group	0.3 mmol/kg/h citrate group	0.6 mmol/kg/h citrate group	1.0 mmol/kg/h citrate group
	Baseline Values			End Values		
pH	7.36 + 0.02	7.36 + 0.03	7.31 + 0.02	7.28 + 0.01##	7.31 + 0.01	7.27 + 0.02##
Base excess [mmol/l]	−0.0 + 0.5	−1.0 + 0.3	−4.8 + 0.5###	−4.2 + 1.0##	−3.8 + 0.9##	−5.4 + 0.5###
pCO_2_ [mmHg]	45.2 + 1.8	43.9 + 3.9	42.0 + 2.2	47.3 + 2.4	43.9 + 2.2	46.2 + 3.4
pO_2_ [mmHg]	437.5 + 12.7	446.7 + 10.0	452.2 + 12.8	444.7 + 11.5	439.3 + 5.9	437.2 + 12.0
K^+^ [mmol/l]	5.1 + 0.1	4.4 + 0.1	6.1 + 0.1###	6.4 + 0.1###	6.0 + 0.1###	5.8 + 0.1###
Cl^-^[mmol/l]	111 + 1	114 + 1	115 + 1	113 + 2	112 + 2	110 + 1**
Na^+^ [mmol/l]	139 + 1	141 + 1	140 + 1	139 + 1	141 + 0	143 + 1**
Glucose [mg/dl]	172 + 14	136 + 5	150 + 6	162 + 26	135 + 9	126 + 12
Lactate [mmol/l]	0.97 + 0.13	0.72 + 0.05	0.68 + 0.07	0.63 + 0.05	0.65 + 0.03	0.62 + 0.08

Values are means ± SE; *n* = 6 per group. Baseline values were obtained from the normoxic control group after the acclimation period; these values were not significantly different from the baseline values of the other experimental groups. The other values were assessed from the last blood sampling at the end of the full ischemia/reperfusion period. For experimental details, see Fig. 1

## *p* < 0.01 and ### *p* < 0.001 vs. normoxic control group. ** *p* < 0.01 vs. I/R control group

Infusion of citrate at any concentration did not change pre-ischemic values of any parameter other than sodium. Sodium levels were higher, but still within the physiological range, in the 0.6 mmol/kg/h and 1.0 mmol/kg/h citrate groups at some time points compared to the I/R control group with a maximum deviation of means of 4.5 mmol/l. Sodium levels showed no significant differences in the follow-up study despite infusion of concentrated NaCl. In the 1.0 mmol/kg/h citrate group and chloride with a baseline value of 112.1 mmol/l was decreased at the end of the reperfusion period (115.2 ± 0.8 mmol/l vs. 109.7 ± 1.2 mmol/l; *p* < 0.01 vs. I/R control group) (Table 1 and Additional file 1: Figure S1). Base excess was significantly lower in this group after 60 min of reperfusion (−6.9 ± 0.6 vs. −3.7 ± 0.4; *p* < 0.01 vs. I/R control group) with no differences being detectable at the end of the experimental procedure (Table 1).

**Fig. 7:**
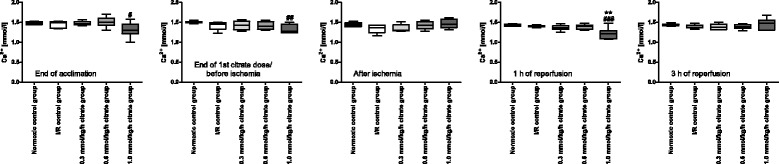
Ionized plasma calcium levels. Box plots with whiskers indicating minimum and maximum, six rats per group. # *p* < 0.05, ## *p* < 0.01 and ### *p* < 0.001 vs. normoxic control group. ** *p* < 0.01 vs. I/R control group

Ionized calcium levels after acclimation ranged between 1.00 and 1.71 mmol/l with a mean of 1.45 ± 0.13 mmol/l. The lowest ionized calcium level observed during the experimental procedure was 1.09 mmol/l (1.0 mmol/kg/h citrate group; 1 h of reperfusion), while the highest level observed was 1.68 mmol/l (1.0 mmol/kg/h citrate group; 3 h of reperfusion). Ionized calcium levels were temporarily lower in the 1.0 mmol/kg/h citrate group following the periods of citrate infusion, but stayed within the baseline range (Fig. 7). Prior experiments in normoxic animals showed that no effects on hemodynamics or any of the measured serum parameters could be detected as long as ionized calcium levels were kept above 1 mmol/l (data not shown).

### Effects of citrate on urine output

Urine output averaged 8.1 ± 1.3 μl/min in the normoxic control group and was stable throughout the experiment. Inter-individual differences ranged from 3 to 17 μl/min (Additional file 2: Figure S2A) and did not correlate with time of measurement. I/R did not have a significant effect on the mean values compared to the normoxic control group. However, following ischemia half of the animals developed a decrease in urine output while the others became polyuric [1 μl/min vs. 27 μl/min] (Additional file 2: Figure S2B). Infusion of citrate did not change this pattern compared to the I/R control group.

### Citrate levels in plasma and urine

**Fig. 8:**
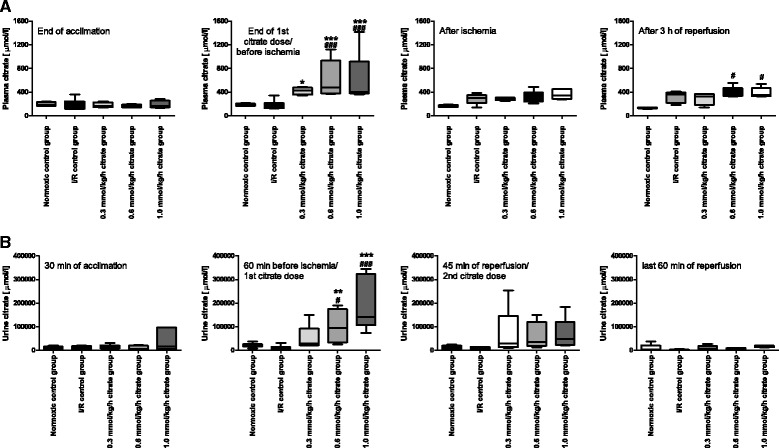
Plasma and urine citrate levels. Box plots with whishers indicating minimum and maximum, 4–6 rats per group. **a** Plasma citrate levels measured at indicated time points. # *p* < 0.05 and ### *p* < 0.001 vs. normoxic control group. * *p* < 0.05 and *** *p* < 0.001 vs. I/R control group. **b** Urine citrate levels measured in samples collected during the indicated time periods. # *p* < 0.05 and ### *p* < 0.001 vs. normoxic control group. ** *p* < 0.01 and *** *p* < 0.001 vs. I/R control group

Citrate levels in plasma were measured 30 min after catheter insertion, immediately before induction of ischemia after administration of the first citrate dose, at the end of 40 min of ischemia and at 180 min of reperfusion. In the normoxic control group citrate levels in plasma decreased slightly during the course of the experiment (197 ± 17 vs. 136 ± 5 μmol/l; *p* < 0.05). Ischemia tended to increase plasma citrate levels, but did not reach significance (Fig. 8a). Citrate infusion increased pre-ischemic citrate levels in plasma compared to the I/R control group.

In the normoxic control group citrate levels in urine did not change during the course of the experiment. Citrate excretion in the I/R control group did not differ from the normoxic control group during any of the indicated time spans (Fig. 8b). Pre-ischemic citrate infusion increased urinary citrate levels in a dose dependent fashion reaching significance in the 0.6 mmol/kg/h (*p* < 0.01) and 1.0 mmol/kg/h (*p* < 0.001) citrate groups compared to the I/R control group. The second, post-ischemic citrate infusion did not significantly increase urinary citrate levels and levels had equalized in all groups by the end of reperfusion.

### Effects of citrate on osmolality

Blood for determination of osmolality was drawn in an additional experiment that compared an I/R control group and I/R 1.0 mmol/kg/h citrate group as described above.

After 60 min of citrate infusion at a rate of 1.0 mmol/kg/h plasma osmolality directly measured by cryoscopy was not different from the plasma osmolality of the I/R control group (301 ± 0.3 mosmol/kg vs. 303 ± 4 mosmol/kg), nor could any difference be detected after 100 min of reperfusion where MAP of the 1.0 mmol/kg/h citrate group peaked (304 ± 4 mosmol/kg vs 306 ± 4 mosmol/kg).

## Discussion

Intravenous infusion of 1.0 mmol/kg/h trisodium citrate in a rat model of AKI (40 min ischemia, 180 min reperfusion) showed protective effects resulting in a small but robust amelioration of increased pCrea and LDH levels and a tendency for higher tissue ATP content. Intravenous infusion of trisodium citrate was accompanied by a concentration-dependent improvement of hemodynamic stability during bilateral renal ischemia. A follow-up study conducted to control for effects of the Na^+^ load going along with citrate confirmed that the protective effects were citrate-dependent.

In humans as well as in rodents trisodium citrate is converted to citric acid which is metabolized in the citric acid cycle at high rates mainly in the liver and to a small percentage also in other organs such as skeletal muscle and kidney [18]. Complete metabolism results in degradation to carbon dioxide and water. As hydrogen is consumed during this process blood pH increases with the potential risk of metabolic alkalosis. On the other hand, an insufficient metabolism might result in citrate accumulation and acidosis. With degradation of citrate the chelated calcium is liberated again.

Citrate is nowadays frequently used for regional anticoagulation where it reduces bleeding risks and appeared particularly beneficial after surgery, in sepsis and severe multiple organ failure showing a positive impact on morbidity, mortality and renal recovery possibly due to interference with inflammation [9]. Additive effects such as reduction of oxidative stress and inflammation have also been reported [11]. Addition of citrate as a citric acid cycle metabolite during reoxygenation of hypoxic proximal renal tubules previously exposed to ischemia improves energetic function and recovery probably by driving substrate-level phosphorylation and decreasing accumulated non-esterified fatty acids [12, 13]. Reduction of the mitochondrial and thereby cellular damage during ischemia and early reoxygenation should possibly enhance cellular resistance to subsequent damage induced by oxygen radical formation and inflammatory reactions occurring in the later course of reperfusion.

To the best of our knowledge, direct renal protection from I/R-induced renal injury by intravenous citrate has not been studied previously.

No adverse effects following citrate infusion were observed in the present study. Ionized calcium levels required for hemodynamic stability were kept within the physiological range [19, 20] by infusion of 10% calcium gluconate at fixed rates.

We utilized a novel intensive monitoring protocol that can uncover central hemodynamic alterations contributing to injury. As observed before, bilateral renal clamping leads to a drop in MAP in our rat model of I/R-induced AKI [14] that persists despite the use of bolus injections of saline to compensate. Induction of hypotension is only observed in bilateral and not in unilateral atraumatic clamping in our model (data not shown). A significant decrease in blood pressure associated with bilateral (and not unilateral) renal ischemia has been described before [21], although to our knowledge studies performing close continuous arterial blood pressure monitoring especially during the clamping period are missing. The mechanism involved in induction of hypotension during bilateral renal clamping remain unclear, but it is conceivable that this effect is related to interference by the clamping itself with sympathetic nerves surrounding the renal artery, an effect similar to that seen with renal denervation. Pre-ischemic infusion of citrate had no immediate effect on MAP. However, infusion of citrate prevented the drop in blood pressure during renal clamping reaching statistical significance in the 1.0 mmol/kg/h citrate group in which MAP also tended to be higher during most of the reperfusion period. The stabilizing effect on blood pressure was accompanied by fewer bolus injections and cannot be explained by intravenous application of a hyperosmotic solution as changes in plasma osmolality were neither detected by blood gas analysis nor by cryoscopy and observed effects could not be reproduced by infusion of hyperosmotic NaCl solution at the same volumes.

Application of NaCl 0.9% has been identified as critical. Negative effects of NaCl 0.9% are ascribed to its unphysiologically high chloride content. The high chloride content is associated with an increased risk of hyperchloraemic metabolic acidosis [22–25] and animal studies have suggested that it is the critical determinant for changes in renal blood flow, mediated primarily by effects on afferent and intrarenal arterial vessels [26–29]. Chloride levels of the 1.0 mmol/kg/h citrate group were significantly different compared to the I/R control group only after 3 h of reperfusion, but of questionable physiological implication. In addition, pH was lowest in the 1.0 mmol/kg/h citrate group. Considering the only slight changes in chloride levels and the missing signs of (hyperchloraemic) metabolic acidosis in the groups receiving NaCl 0.9%, it seems very unlikely that observed effects of citrate can simply be ascribed to a reduced application of NaCl 0.9% in the 1.0 mmol/kg/h citrate group.

Ionized calcium levels observed during the experimental period were never outside the baseline range. It is unlikely that reduced ionized calcium levels as observed in the 1.0 mmol/kg/h citrate group at time points before and after ischemia had any beneficial effects. In addition, ionized calcium levels were not different during ischemia in all groups when differences in blood pressure occurred between groups. Minor, although significant differences observed at other time points were not accompanied by hemodynamic distinctions.

Even before induction of ischemia urine output was very variable. Variability increased after induction of ischemia ranging from anuria to polyuria. Urine output was not correlated to changes in pCrea, LDH concentrations or tissue ATP content. Clamping of the renal pedicle should convey a homogeneous burden of injury to all animals. Temporary polyuria as a sign of tubular malfunction secondarily turning into oliguria/anuria has been described in rats [30] and is a common feature in (human) patients, too. Urine output is an observational parameter only. Although oliguric AKI has a worse prognosis than non-oliguric AKI [31], it is unclear in how far urine output directly following hypoxia might reflect the severity of renal injury or differences of such.

Since citrate is delivered as trisodium citrate, infusion of each molecule of citrate is accompanied by three ions of sodium. Therefore, the 1.0 mmol/kg/h citrate group received 3.0 mmol/kg/h sodium during 60 min prior and 45 min post ischemia compared to 0.77 mmol/kg/h in animals infused with NaCl 0.9%. There is a possibility that the extra sodium increases intravascular volume and thereby favors better early reperfusion and intratubular solute flow with amelioration of I/R-induced injury. Changes in plasma sodium levels did not strictly correlate with citrate infusion periods and were minimal. In addition, 60 min of citrate infusion before renal clamping did not result in increased blood pressure making large changes of intravascular volume due to sodium load less likely. Nevertheless, a follow-up study conducted to control for high sodium load accompanied by infusion of citrate confirmed that protective effects were produced by citrate rather than by sodium. Further increase of citrate delivery to 1.5 mmol/kg/h did not result in additional improvement (data not shown).

Baseline citrate levels in plasma were in the range of those observed for adult Sprague Dawley rats [32]. Ischemia itself tended to increase plasma citrate concentrations compared to the normoxic control group, while citrate infusions resulted in pre-ischemic elevated plasma citrate levels which seemed to saturate at maximum concentrations of about 600 μmol/l. The infused citrate increased urinary excretion indicating substantial delivery to the kidney, but underwent rapid extrarenal metabolism and clearance as indicated both by the saturation during infusion and the sharp decrease of plasma citrate during the ischemia clamp period when citrate was not being infused and could not be cleared by the kidneys.

Urinary citrate excretion was increased in all animals receiving citrate during the pre-ischemic period compared to the normoxic and ischemia control groups reaching maximum urinary levels of approximately 220 mmol/l in the 0.6 and 1.0 mmol/kg/h citrate groups. At the end of the experiment plasma citrate levels of the citrate-infused animals were no different from the levels of the I/R control group and post-ischemia citrate excretion did also not differ between groups.

An ischemia-induced increase in citrate has been demonstrated after myocardial ischemia in the porcine heart, where it was suggested to inhibit glycolysis and thereby reduce the heart’s ability to compensate for energetic deficiency [33]. Because of changes in myocardial net release, citrate has been discussed as a potential marker of myocardial ischemia [34]. As citrate is freely filtered by the glomeruli, urinary citrate excretion is mainly influenced by the rate of proximal tubular citrate reabsorption through a Na+/citrate transporter located in the apical membrane [35, 36]. Urinary citrate excretion has been identified as a marker of favorable outcome after autotransplantation of kidneys following cold storage in pigs [35]. It was suggested that a reduction in urinary citrate excretion is due to the impairment of oxidative energy metabolism promoted by I/R-injury. In our model urinary citrate excretion is primarily determined by infused citrate. Nevertheless, a reduction of urinary citrate might indicate a lack of its production in the citric acid cycle and supplementation of citrate can help to restore metabolic function.

Pre-ischemic enhanced glomerular filtered citrate reabsorbed by the proximal tubules representing the vulnerable site of the kidney may increase the intra-tubular citrate concentration and offer benefit by driving substrate-level phosphorylation for anaerobic ATP production during the reperfusion period. Inhibition of glycolysis by citrate as opposed to myocardial cells [33] is not an issue in renal proximal tubular cells which have very little glycolytic capacity [37]. Citrate supplementation of isolated proximal tubular cells during hypoxia and reoxygenation, but not during hypoxia alone, improved mitochondrial membrane potential and ATP levels. Under aerobic conditions, as present during reoxygenation, citrate may promote forward operation of the citric acid cycle resulting in α-ketoglutarate production to drive the substrate-level phosphorylation rescue pathway. Direct application of α-ketoglutarate showed adverse effects in our model as systemic effects on hemodynamics compete with tubular effects [14].

Hemodynamic stabilization during ischemia by citrate could improve outcome by favoring early reperfusion and limiting the important damage that occurs at that time. It is notable in this regard, that α-ketoglutarate/malate in our prior study of this type worsened hypotension during ischemia and did not show the protection seen with citrate even though it protects isolated tubules better than citrate and, unlike citrate, is effective when administered to them during hypoxia only [12, 14].

Citrate infusion in our rat model of I/R-induced AKI did not affect the histological changes seen at the 3 h reperfusion time point studied. However, these changes in the outer cortex are known to be highly reversible and do not allow any conclusions with regard to the more severe outer medullary damage that only develops later (at least 6–12 h following the initial event) and is probably more functionally significant. Information about structural protection is, therefore, limited by the time frame of the model.

Patients afflicted by AKI especially in the intensive care setting are often exposed to many, repeated and/or ongoing incidences of damage (drops in blood pressure, toxic events, etc.). Apart from renal replacement therapy, where currently used concentrations might be too low, citrate could be considered as a protective agent itself. It is unclear, if supplementation of citrate after ischemia only provides any protective effect as this was not tested in our experimental setting. In clinical settings involving continuous renal replacement therapy citrate could be protective in cases of repeated and/or ongoing incidences of damage.

## Conclusions

In summary, we have applied a novel approach with intensive biomonitoring to study the effects of sodium citrate infusion with calcium support on I/R-induced AKI. Citrate itself was well tolerated, appeared in the urine, and also underwent rapid extrarenal metabolism. Citrate infusion at 1.0 mmol/kg/h showed renal protection during the early post-ischemic phase. This effect may derive both from the benefits of citrate for tubule cell metabolism that have previously been observed in isolated tubules as well as from citrate-associated prevention of systemic hypotension that occurs during the ischemic period. The data support the rationale for further investigation of citrate infusion as a therapeutic modality.

## Additional files

Additional file 1: Figure S1. Ionized plasma chloride levels. Box plots with whiskers indicating minimum and maximum, six rats per group. ** *p* < 0.01 vs. I/R control group. (PDF 30 kb)

Additional file 2: Figure S2. Urine output. Urine output calculated from samples collected during *A* 45 min before ischemia *B* 120–180 min of reperfusion. (PDF 22 kb)

## Abbreviations

AKI: Acute kidney injury
ATP: Adenosine triphosphate
FELASA: Federation of European Laboratory Animal Science Association
I/R: Ischemia and reperfusion
KDIGO: Kidney Disease Improving Global Outcomes
LDH: Lactate dehydrogenase
MAP: Mean arterial blood pressure
pCrea: Plasma creatinine
