# Supplementary figures and images for: Citrate shows protective effects on cardiovascular and renal function in ischemia-induced acute kidney injury
Source: BMC Nephrol. 2017 Apr 10;18:130. doi: 10.1186/s12882-017-0546-1 (PMC5387390; doi:10.1186/s12882-017-0546-1)

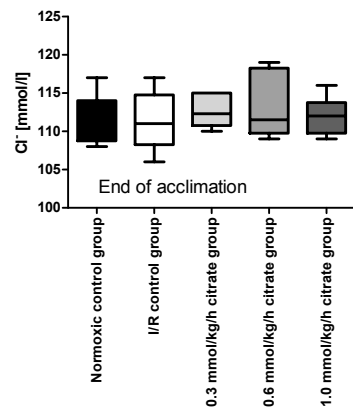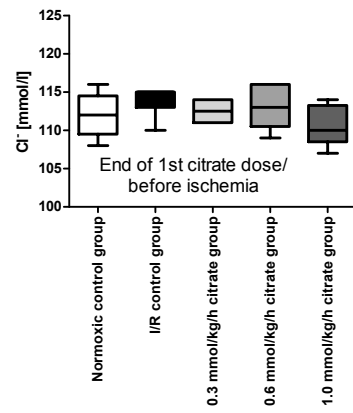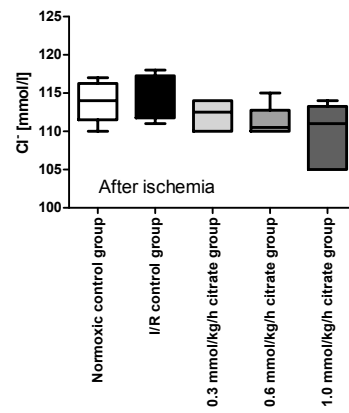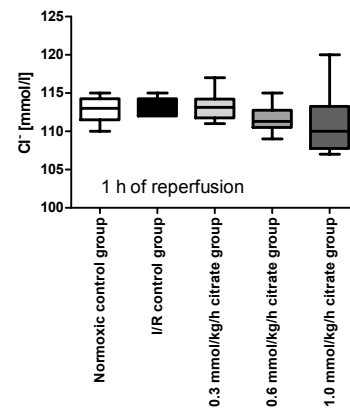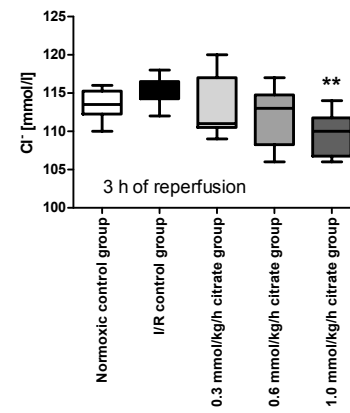

Supplement: Supplementary file 1 — Ionized plasma chloride levels. Box plots with whiskers indicating minimum and maximum, six rats per group. ** p < 0.01 vs. I/R control group. (PDF 30 kb) [file 12882_2017_546_MOESM1_ESM.pdf]

**A**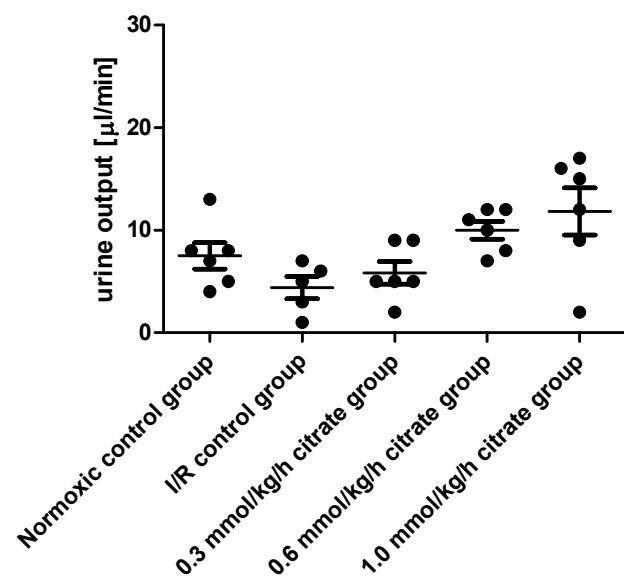**B**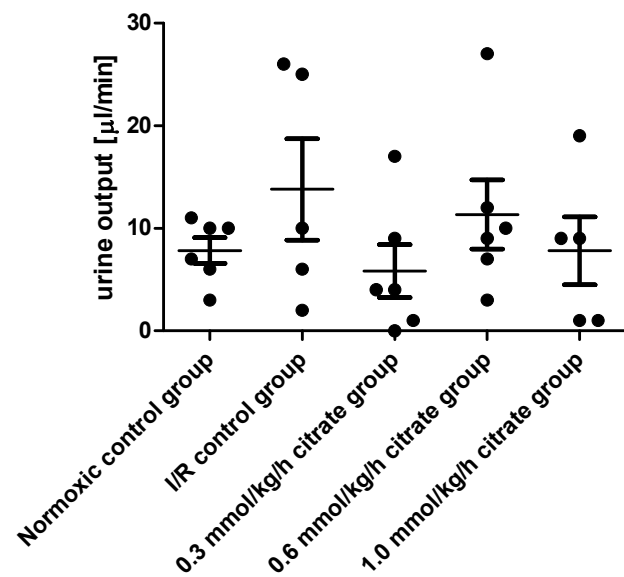

Supplement: Supplementary file 2 — Urine output. Urine output calculated from samples collected during A 45 min before ischemia B 120–180 min of reperfusion. (PDF 22 kb) [file 12882_2017_546_MOESM2_ESM.pdf]
